# Estimation of the current healthcare costs of gout in Liaoning Province from 2015 to 2022 based on the SHA2011 accounting system

**DOI:** 10.3389/fpubh.2025.1646950

**Published:** 2025-09-18

**Authors:** Xiaoxia Shi, Fengchun Zheng, Quan Wan, Peipei Chai, Guilin Wang, Zhenmiao Pang, Yuedan Ma

**Affiliations:** ^1^School of Public Health and Management, Guangzhou University of Chinese Medicine, Guangzhou, China; ^2^Liaoning Health Economics Association, Shenyang, China; ^3^China National Health Development Research Center, Beijing, China; ^4^Department of Traditional Chinese Medicine, School of Graduate Students, Liaoning University of Traditional Chinese Medicine, Shenyang, China; ^5^Department of Public Management, School of Economics and Management, Liaoning University of Traditional Chinese Medicine, Shenyang, China

**Keywords:** gout, direct medical expenditure, medical service utilization, SHA2011, social health insurance

## Abstract

**Background:**

Gout has become a major public health problem worldwide, causing severe pain, discomfort, inflammation, mobility problems and impaired physical functioning, resulting in heavy economic losses and social burdens.

**Methods:**

The study was based on System of Health Accounts 2011 (SHA2011) and data of 97,907 patients from 1,084 healthcare organizations were taken using multistage stratified random sampling method. Descriptive analysis of therapeutic care expenditures (CCE), Sankey diagram to analyze the flow of CCE, Mann–Whitney U test to analyze 2 independent samples, Kruskal-Wallis H to analyze K independent samples significance. Multifactorial analysis of CCE influences, Structural Equation Modeling (SEM) to analyze the direct and indirect effects and mediating role.

**Results:**

CCE increases from CNY 25.32 million in 2015 to CNY 116.02 million in 2022, with a concentration of ages 30–69. The proportion of public health financing decreases with age and is dominated by general hospitals. The difference is significant in single-factor analysis, and the high cost in multi-factor analysis is related to purchase drugs, elective treatment, basic medical insurance for urban and rural residents, provincial level, Chinese medicine hospitals, and large standardized coefficients in 2020, and outpatient/inpatient care, drugs, type of institution, insurance status, and gender by path coefficients in the SEM in the order of directly or indirectly affecting the CCE.

**Conclusion:**

Gout disease in Liaoning Province imposes a heavy financial burden on patients and the health insurance system. It is recommended to increase reimbursement for gout inpatient costs, improve primary care guidelines and purinol dose adherence, strengthen primary care collaborative personalized education and care to achieve uric acid-lowering effects, increase subsidies and inclusion of gout prescription medications in the local health insurance directory, and for men to reduce consumption of high-purine foods and alcohol at social events.

## 1 Introduction

Due to the accumulation of urate crystals in the joints, gout causes highly intense pain, lingering discomfort and inflammation, and limited range of motion at the joints, it has become a common metabolic disease after diabetes (1) and carries a heavy social burden (2). Worldwide prevalence is increasing, parallel to the increase in non-communicable diseases (3). Nearly 56.47 million people suffer from gout globally in 2021 (4), and the prevalence of gout is around 1–6.8% (5), with the USA having a prevalence of gout of 3.9% (10.50 million) (6, 7), Germany having the highest prevalence of 1.64% (1.33 million), Taiwan 2.21% (0.50 million), UK 2.5% (0.95 million) (8), Greece 4.75% (0.17 million) (9), Australia 5.2% (0.75 million), France 1.47% (0.92 million), Canada 3.37% (1.19 million), Italy 1.48% (0.86 million) and New Zealand 3.09% (0.15 million) (4). The global disability-adjusted life years (DALYs) due to gout in 2021 were 2,484,546 in the United States (358545), Germany (46466), Taiwan (18420), Canada (40146), Italy (29662), and New Zealand (5191) (4), with the highest age-standardized percentage of DALYs observed in the Western European countries (including the Nordic countries) (10).

Gout leads to mobility problems and impaired physical functioning, gout also imposes financial costs on the patient (11, 12), gout induced increase in healthcare expenditures (13). An untreated gout places a heavy load on the global health system as chronic gout leads to gout development, persistent articular pain, joint erosion and injury, contributing to increased rates of morbidity and DALYs (6, 14), all of which contribute to higher mortality rates (15). In addition to the added personal economic cost, there are yearly consequences of direct and indirect economic losses exists for those who do not manage their gout well (16), with gout related costs, both direct and indirect, ranging from nearly $4,000 to $18,000 per capita globally in 2017 (17). Indirect costs for gout patients in the United States were £2,883 from 2001 to 2004 (18), the direct and indirect costs of gout in the United States are estimated to be over $6 billion in 2021 (19, 20), and the total all-cause cost of care is $11,663 per person or $31.8 billion total (21), and in the United States, based on different employment (18), older adults (22), and refractory gout (23) populations the per capita cost of treating gout disease was $4,733, $16,925, and $18,362, and a certain study in Western Sydney in 2017–2018 showed 472 patients with 552 emergency department visits for treatment of gout at a cost of AUD$367,835, and 310 admissions to hospitals with gout as the main diagnosis at a cost of AUD$1,730,000 (24). The average per capita medical cost for gout patients in Canada was $134 per month, with a total of $8,020 per patient for treatment over 5 years (25), with an incremental cost of $1,861 in the province of Ontario, Canada (26, 27), and a total medical cost of $44,297 for gout patients over 5 years (27). In Australia, where the median overall direct cost of gout equaled A$200 for a patient per year, the three categories contributing most to expenditures were prescription medications (A$207), over-the-counter medications (A$87), with herbal traditional medicines (A$84), which represented 57%, or A$378, of the mean overall direct cost (28). The average direct total cost of gout in Spain was €2,228 and the average indirect total cost was €68.37 (29), and for gout patients in Spain from 2003 to 2007 the total cost was €7 million, with 96.9% of direct medical expenses while 3.1% were non-medical expenses (productivities lost) (29). Studies estimating gout-related charges as a result of healthcare claims have reported values ranging from $332 (30) to $9,748(31) and $12,620 (26). Frequent-onset gout in Taiwan has a median cost of US$369 (31), and the average annual healthcare expenditure for older adults gout patients in the general population in Taiwan was US$1461.2, with outpatient treatment costing US$1032.5 and inpatient hospitalization costing US$428.6 (32); Taiwan’s retrospective database analysis conducted on the basis of the 2010 Longitudinal Health Insurance Database (LHID) yielded a Gout mean incremental and all-cause healthcare costs of US$1551 and US$2985 (31). In the Netherlands, Spaetgens et al. (33) estimated in just one recent study the combined yearly direct and indirect (excluding attendance) costs for the whole population to be €6,914 for each patient annually, the yearly direct costs (€5,647 for each patient annually), with a direct cost share of 82%, and the cost of gout-induced absenteeism to be €4,982.

According to a recent survey of thirty-one different provinces in continental China, gout has become a major public health problem in China, the loaded rate was 3.2% for adult gout in China, and it is estimated that by the year 2021 to 100 million people in China will suffer from gout (34), with prevalence rates of gout ranging from approximately 0.83–1.98% in males, and 0.07% −0.72 in females (35), and mean age of the victims is (48.80 ± 15.10) years old (36), with males 47.95 years, and 53.14 years for women (37); Qingdao, China, from 0.36% in 2002 to 0.53% in 2004 (38); Shandong Province, from 0.50 to 2.55% (39); Beijing, 0.09% (40); Ningbo, Zhejiang Province, 0.29% (41); and Qiang, Beichuan County, Mianyang City, Sichuan Province, 0.92%, male 1.37 and 0.4% for females (42); Zhangjiakou City, Hebei Province, workers as prevalence 1.2% for males and 0.3% for females (43); Nanjing City, Jiangsu Province, 1.33% for males, 1.98% for males and 0.72% for females (44); and Shandong Province, coastal areas, prevalence 1.36% (45). The prevalence in Shenzhen City was 4.6, 4.11% for men and 0.63% for women (46); in Huiyang District, Huizhou City, the prevalence was 1.5%, in male and 0.01% in female (47); and the overall morphology rates of gout among the Brown ethnic group in the Brown Mountain area of Yunnan Province were 9.39, 16.13% in male and 4.42% in female (48). The per capita cost of regular medication for 1 year for gout patients in the First Hospital of Nanjing is 8414.71 yuan, and the total cost per capita for patients who hardly take gout medication is 8162.23 yuan (49); the total cost of anti-gout medication in 2021 for the People’s Hospital of Macheng City, Hubei Province, is 169,210.43 yuan, with the highest average daily cost of 18.74 yuan (50). The median total hospitalization cost for gout in Guangzhou city’s health insurance decreased from 8295.23 yuan (2017) to 7270.70 yuan (2019), the median out-of-pocket amount increased from 2158.45 yuan (2017) to 2660.35 yuan (2019), and the median cost of the comprehensive medical category increased from 721.83 yuan (2017) to 807.49 yuan (2019) (51).

In gout studies, all have used the Wilcoxon signed rank sum test primarily for continuous variables and the χ^2^ test for categorical variables in data on gout costs and demographic characteristics (23, 52), and some studies have used the t-test to compare differences in costs (18, 25, 52, 53), and some studies in particular have utilized the Wilcoxon rank sum (Mann–Whitney) test and continuity-corrected Pearson χ^2^ to test for differences in median gout attack costs, respectively (52), and McNemar’s test for categorical variables (26). The most commonly used two-part model for gout cost estimation is for estimating gout costs only is in the first part, logistic regression was used to predict the likelihood of in-year medical costs being greater than zero, and in the second part, a generalized linear model was used to estimate the average annual medical costs of employees with positive medical costs, and the results were combined to estimate annual medical costs for all employees (18); for the group with gout and the no-gout group as a control is, the first step used the no-gout group and the no-gout group as a control. As a control is, the first step uses a two-part model for gout-free, the first part is a logistic regression model that predicts whether or not any costs are incurred, and the second part is a generalized linear model (GLM) with a log link function and a gamma distribution to determine the amount of costs, which generates a predictive model for gout-free costs; in the second step, the coefficients from the predictive model are used to estimate the costs for each patient with gout, using the Bootstrap resampling method to estimate differences in healthcare costs (26); for refractory gout, Poisson regression was performed using a 2-part logistic/generalized linear model with a gamma distribution for emergency visits, ER visits, hospital days, and productivity, and healthcare costs were analyzed using logistic regression for the variables that were correlated with the presence of ≥3 gouty outbreaks (the dependent variable) (Statistics Wald) (29), and the cost impact of gout attack frequency was estimated using repeated measures 2-part regression modeling (53). Gout has shifted from being a disease associated with the affluent to affecting the older adults (54, 55). Furthermore, the important role of genetic factors in the development of gout (56) and mutations in specific genes are strongly associated with gout risk (57). Triglyceride/high-density lipoprotein (TG/HDL) ratio is associated with the incidence of gout (58). Fructose intake is directly or indirectly associated with gout, and fructose increases the occurrence of metabolic syndrome (59).

SHA2011 is an internationally recognized scientific method and systematic tool for tracking the entire flow of health funds from source, to flow, to use, and is an important method for identifying the health care burden of the population and the degree of risk protection, and is currently the most advanced international health cost accounting method (60, 61). Gout has become a major public health problem in China (62), and any change in the overarching burden may inform health policy makers’ decisions regarding the prevention and screening of gout. Therefore, updated data based on SHA2011 accounting for the gout burden will contribute to improving this disease’s management.

## 2 Methods

### 2.1 Data source

**Figure 1:**
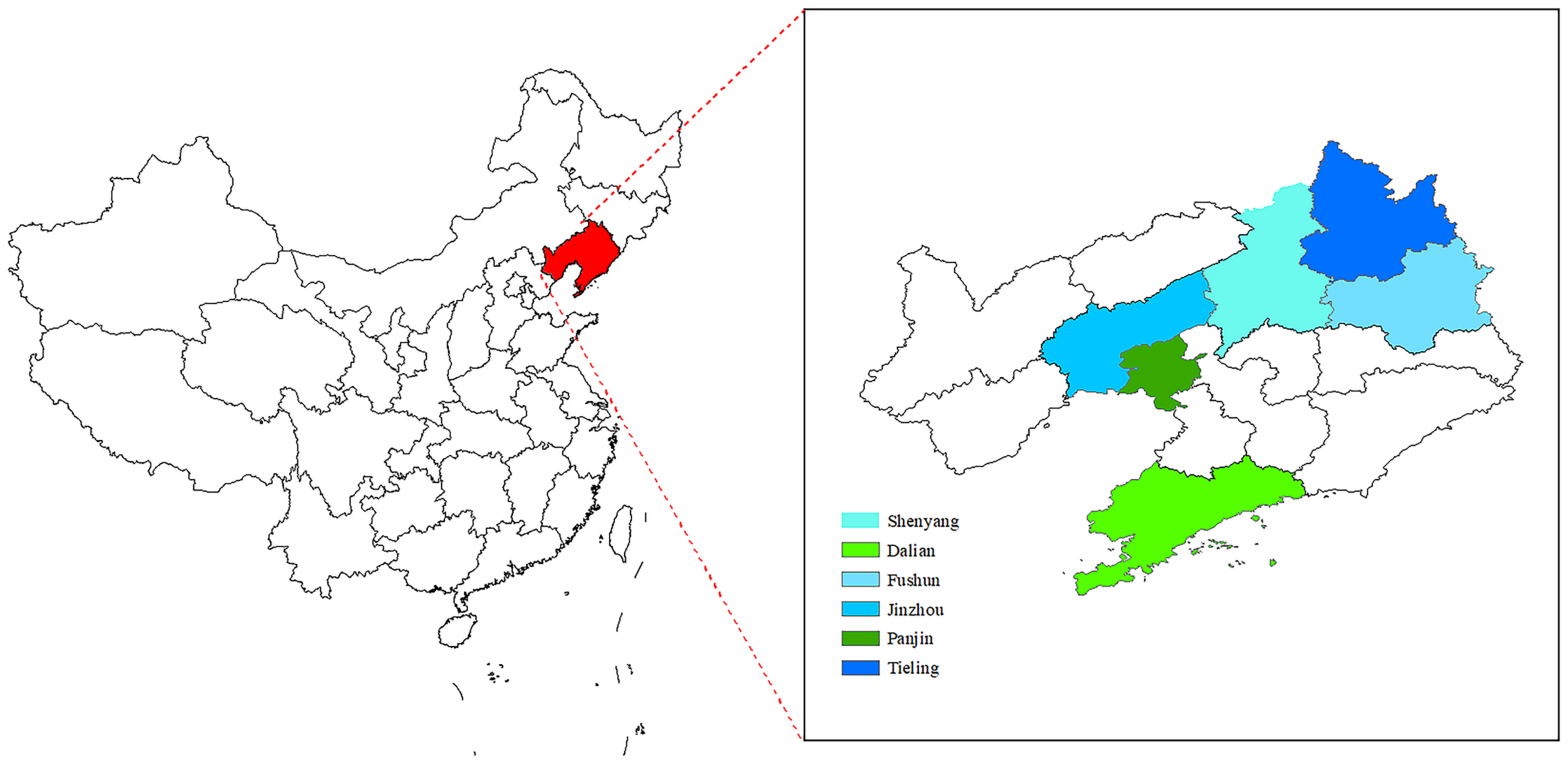
Map of provinces and cities studied.

The macroeconomic indicators of Liaoning Province utilized in this study include the 2015–2022 Liaoning Health Statistics Yearbook, the Liaoning Health Financial Annual Report, the Liaoning government health input monitoring table, the social decision table of the Liaoning Provincial Department of Finance, the breakdown of various types of health insurance of the Liaoning Provincial Health Insurance Bureau, the results of the Liaoning source and institutional methods of measurement, and the Liaoning Provincial Supervisory Bureau’s statistical table of the operation of insurance, which provide great support for the measurement of the Liaoning Province gout. This is a great support for measuring the cost of gout in Liaoning Province. Liaoning Province has 14 cities and municipalities, the overall size of the population is exceptionally large, the overall number of healthcare organizations is exceptionally high, 37,124, involving plus the geographic location is exceptionally wide, so this study adopts a multi-stage sampling method to draw the sample. Multi-stage stratified random sampling saves costs and time, and enhances the typicality and reliability of the sample. According to the Liaoning Statistical Yearbook 2023, the 2022 GDP ranking can be determined that Dalian City ranked 1st, Panjin City ranked 5th, Jinzhou City ranked 6th, Fushun City ranked 9th, Tieling City ranked 13th, 14 cities ranked in the middle and back of the sample, the GDP of the cities and municipalities accounted for 44.04% of the province’s GDP, the representativeness of the city is high; and then according to the geographic location of the city can be seen, Fushun City is located in the east of Liaodong, Jinzhou City belongs to western Liaoning, Dalian City belongs to southern Liaoning, Tieling City belongs to northern Liaoning, and Panjin City belongs to central Liaodong. Then, according to the geographic location, Fushun City is located in Liaodong, Jinzhou City belongs to Liaosi, Dalian City belongs to Liaonan, Tieling City belongs to Liaonan, and Panjin City belongs to Liaonanzhong, which makes the geographic distribution of the sampled cities more comprehensive; in the ranking of the number of permanent resident population in 2022, Dalian City ranked the 2nd, Tieling City ranked the 4th, Jinzhou City ranked the 8th, Fushun City ranked the 11th, and Panjin City ranked the 13th, and the permanent resident population of the sampled cities accounted for 39.56% of the province, which is a strong representation. In 2022, the number of medical and health institutions in Dalian City ranked 3rd, Tieling City ranked 4th, Jinzhou City ranked 8th, Fushun City ranked 11th, and Panjin City ranked 13th, and the number of medical and health institutions in the sampled cities accounted for 34.12% of the province (Table 1). Using a multiphase stratified random sampling method, in the first stage, sample municipalities were selected from 14 municipalities in Liaoning Province, and five selected municipalities, Dalian, Panjin, Fushun, Jinzhou, and Tieling, were determined by ranking of GDP per capita, geographic location, and number of healthcare institutions (Figure 1); in the second stage, the same methodology was applied to sample 1 district and 2 counties according to GDP per capita rankings of the cities, geographic location, and number of healthcare institutions, including Shuangtaizi District, Panjin City, Panjin City, Panshan County, Panjin City, Dawa District, Fushun City, Xinfu District, Fushun City, Fushun City, Fushun City, Xinbin County, Fushun City, Beizhen City, Jinzhou City, Linghai City, Jinzhou City, Guta District, Jinzhou City, Yinzhou District, Tieling City, Tieling City, Tieling County, Tieling City, Kaifuyuan City, Pulandian District, Wafangdian City, Dalian City, and Zhongshan District; at the third stage, 46 general hospitals, 14 medium-sized hospitals, 14 specialized hospitals, 10 women’s and children’s health centers, 3 public medical institutions, 119 commune health centers/community health service centers, and 878 countryside health offices/community health service stations/outpatient units/clinics, for a total of 1,084 medical and health institutions. Various types of healthcare institutions extracted full-year outpatient and inpatient data for measuring therapeutic TCM costs, mainly including patients’ gender, age, diagnosis/admission/discharge department, disease name and ICD-10 code, total fees paid, examination fees, consultation fees, registration fees, bed fees, nursing fees, drug fees (western medicine fees, proprietary Chinese medicine fees, Chinese medicine tablets fees), type of institution, payment method of health insurance and amount, etc. for each patient.

**Table 1:** GDP, resident population, and number of healthcare institutions in 14 cities in Liaoning Province, 2022.

Cities	GDP (CNY million)	Rankings	Resident population (thousand)	Rankings	Number of Health Institutions	Rankings
Shenyang	769580.00	2	6087.00	2	5,103	1
Dalian	843090.00	1	7658.00	1	4,027	3
Anshan	186320.00	3	3306.00	3	2,140	7
Fushun	92770.00	9	1788.00	11	1,353	11
Benxi	93080.00	8	2269.00	9	827	14
Dandong	89070.00	11	1293.00	14	1827	9
Jinzhou	120170.00	6	2273.00	8	1938	8
Yingkou	143160.00	4	1993.00	10	2,301	6
Fuxin	57770.00	14	2703.00	7	1,239	12
Liaoyang	89180.00	10	1701.00	12	1,472	10
Panjin	139430.00	5	1402.00	13	1,155	13
Tieling	75420.00	13	3255.00	4	2,678	4
Chaoyang	99500.00	7	2861.00	5	4,068	2
Huludao	87060.00	12	2805.00	6	2,551	5
Proportion of sampled municipalities in the province (%)	44.04		39.56		34.12	

Sample cities include Dalian, Fushun, Jinzhou, Panjin and Tielin.

### 2.2 Samples

The research samples were patients in Liaoning Province diagnosed with gout disease for the first time from January 1, 2015 to December 31, 2022, and the diagnostic criterion was M10 of the tenth revised edition of the International Classification of Diseases (ICD-10), and a total of 99,055 cases of gout-related diseases were collected. The cleaning of outpatient hospitalization information entries included in the calculation mainly includes gender, which can only appear in codes 1 and 2; age, which cannot have negative values or unreasonable age numbers; disease name and ICD-10 must correspond to the ICD-10 code list one by one, and if the name of the disease appears to be garbled, it should be supplemented correctly according to the ICD-10, and only the name of the disease needs to be supplemented with the ICD-10; the time needs to be accurate only up to Year, month and day, do not need to be accurate to the specific day minutes and seconds, etc.; the type of institution, the type of insurance can only appear in the country issued outpatient hospitalization template labeled consistent; at the same time, for the emergence of health insurance, the integrated fund payment and personal account must have a numerical value, or else the type of health insurance for the out-of-pocket expenses. For the organization level needs to be strictly in accordance with the organization belongs to the provincial urban and county level units to determine. Finally, the total outpatient costs should be consistent with the sum of the outpatient cost details and also with the sum of the funds reimbursed by each type of insurance (63–66). Other exclusion criteria included hospitalization duration of less than 1 day, hospitalization costs of less than 100 RMB (100 RMB 15.3 USD), or missing information (67). Samples with missing or abnormal data, such as age, gender, and medical costs, were excluded (64). A total of 1,148 cases were treated as missing values, and the overall final effective sample size of gout diseases after exclusion was 97,907 cases.

### 2.3 Calculating the CCE process for gouty diseases

SHA2011 accounts for Liaoning Province’s general health costs by estimating the total based on the sample. A basic database of patient visit costs for the sample institutions was established by collecting patient visit cost data from the sample institutions in the sample cities of Liaoning Province. The cost data of nonpublic health institutions were obtained from statistical yearbook of health, and the cost data of public health institutions were obtained from annual report of health finance, which together summed up to the total health cost data of Liaoning Province. The proportion of healthcare expenses of gout patients in the sample to the healthcare expenses of patients with all sample diseases was first calculated, and then multiplied with the total healthcare expenses of Liaoning Province to derive the CCE of gout patients within Liaoning Province.

The gout CCE includes basic expenditure subsidy (BES) and curative income (CI).


$$S_{\text{gCCE}}=S_{\text{gOBES}}+S_{\text{gOCI}}+S_{\text{gIBES}}+S_{\text{gICI}}$$

$\;S_{\text{gCCE}}$ denotes gout disease curative care expenditure, $S_{\text{gOBES}}$ denotes gout outpatient basic expenditure subsidy, $S_{\text{gOCI}}$ denotes gout outpatient curative income, $S_{\text{gIBES}}$ denotes gout inpatient basic expenditure subsidy, $S_{\text{gICI}}$ denotes gout inpatient curative income.

Since gout inpatient CCE is calculated in the same way as outpatient, this study only demonstrates the gout outpatient CCE calculation process.


$$S_{\text{gOCCE}}=S_{\text{gOBES}}+S_{\text{gOCI}}$$

$S_{\text{gOCCE}}\;$ denotes gouty outpatient curative care expenditure (OCCE), gouty outpatient CI, firstly, the outpatient total medical and health institution income of Liaoning province was obtained from the health statistics yearbook and the health finance annual report (OTMI). Then the proportion of outpatient prevention costs was calculated in the outpatient sample base database, and the total outpatient prevention costs in Liaoning Province were obtained by multiplying this proportion by the total outpatient income, and finally outpatient prevention costs were excluded from the overall outpatient income to get the total outpatient CI. Finally, using the whole outpatient income to exclude the total outpatient prevention cost to get the outpatient CI.


$$S_{\text{gOCI}}=S_{\text{gOTMI}}\times (1-\frac{\alpha_{p}}{\alpha })$$

$\;S_{\text{gOTMI}}\;$ denotes gout outpatient total medical and health institution income, $\alpha_{p}$ denotes sample institution outpatient total preventive costs, calculated by summing the costs of all services involving preventive visits in the sample institution. α denotes sample institution outpatient total income.

The formula for calculating the gout outpatient CI for a particular dimension is as follows:


$$S_{\text{gOCI}}^{\prime }=\sum_{i=1}^{n}(S_{\text{gOCI}}\times \frac{\alpha_{i}}{\alpha -\alpha_{p}})$$

$S_{\text{gOCI}}^{\prime }$ denotes gout outpatient CI under a certain dimension, $\;\alpha_{i}$ denotes outpatient CI for a particular patient visit in the sample. Gout outpatient basic expenditure subsidy (OBES), total outpatient basic expenditure subsidy (TOBES), total inpatient bed days (a), and number of outpatient visits (b) in Liaoning province were first obtained from the combined health statistics yearbook and health finance annual report. The ratio of the number of visits involving preventive services (c) to the number of all visits in the sample (d) was then eliminated from the sample organizations and the number of visits (b) was applied to obtain the number of therapeutic outpatient visits in Liaoning Province (e). Correlation of therapeutic outpatient visits to total inpatient bed days was used as the apportionment coefficient (*β*).


$$S_{g\text{OBES}}=S_{\text{TOBES}}\times (1-\beta )$$

$\;S_{\text{TOBES}}$ denotes Liaoning Province total outpatient basic expenditure subsidy.


$$\beta =\frac{\alpha }{\alpha +e\times K}$$

K is the conversion relationship between a physician’s workload of one inpatient bed day and one outpatient visit by the National Center for Health Development Research (NCHDR) by investigating the relationship between the workload of a physician as one inpatient bed day and one outpatient visit, resulting in K = 0.1.


$$e=b\times (1-\frac{c}{d})$$

The formula for calculating the OBES for gout in a particular dimension is as follows:


$$S_{\text{gOBES}}^{\prime }=\sum_{i=1}^{n}(S_{\text{gOBES}}\times \frac{\alpha_{i}}{\alpha -\alpha_{p}})$$

The follow-up of this study according to different types of healthcare organizations, different levels of healthcare organizations, and age groups were all calculated according to this formula.

### 2.4 Factors affecting the expenditure of gout disease

Descriptive statistics were used in this study for different subgroups, and because the cost data were positively skewed, costs for gout disease were described by intermediate and interquartile range (IQR). We performed univariate analysis with Mann–Whitney U test as well as Kruskal-Wallis H test to determine the significance of within-group differences in gout costs, and two-by-two comparisons of three and more categorical variables that were significant to further determine the significance of within-group differences in three and more categorical variables. Categorical variables with significant results were first set as dummy variables and then included in multiple linear regression analyses to analyze variables that have a large influence on gout disease. Further, the direct and indirect effects of different variables on the cost of gout disease were analyzed by constructing structural equation models. All comparisons were analyzed using STATA 15.0, SPSS 25.0, AMOS 20.0 (SPSS) statistical software and statistical significance of all comparisons had a *p* value <0.05.

## 3 Results

According to the findings in Table 2, the CCE of gout disease in Liaoning Province rose with a growth rate of 358.21% from CNY 25.32 million in 2015 to CNY 116.02 million in 2022, with more inpatient costs, which grew from CNY 11.95 million in 2015 to CNY 91.14 million in 2022, representing a growth rate of 662.68%, and outpatient costs are less, growing from CNY 13.37 millions of 2015 to CNY 24.87 millions of 2022, at a growth rate of 86.01%. The cost of rheumatic diseases rose from CNY 811.07 million in 2015 to CNY 2099.81 million in 2022, a growth rate of 158.89%, with higher inpatient cost, which rose from CNY 480.72 million of 2015 to CNY 809.26 million of 2022, a growth rate of 68.34%, and outpatient cost, which rose from CNY CNY 330.34 million to CNY 1290.55 million in 2022, an increase of 290.67%. Gout disease is a type of rheumatoid immune disease, the share of which grows from 3.12% in 2015 to 8.33% in 2017, then decreases and picks up again to 9.35% in 2020, gradually decreasing to 5.53% in 2022.

**Table 2:** Gout CCE in Liaoning Province, 2015 to 2022 (CNY million).

Year	Gout health CCE	Outpatient	Inpatient	Total Rheumatic Immunological Diseases	Outpatient	Inpatient	Gouty diseases as a proportion of rheumatic diseases (%)
2015	25.32	13.37	11.95	811.07	330.34	480.72	3.12
2016	52.08	16.74	35.34	1279.53	632.67	646.87	4.07
2017	89.42	39.99	49.43	1072.85	325.87	746.98	8.33
2018	98.27	41.00	57.27	1296.61	695.97	763.74	7.58
2019	107.46	48.89	58.57	1365.35	550.53	814.82	7.87
2020	95.95	34.63	61.33	1025.91	527.22	498.69	9.35
2021	106.77	29.19	77.58	1403.46	476.19	927.27	7.61
2022	116.02	24.87	91.14	2099.81	1290.55	809.26	5.53

**Figure 2:**
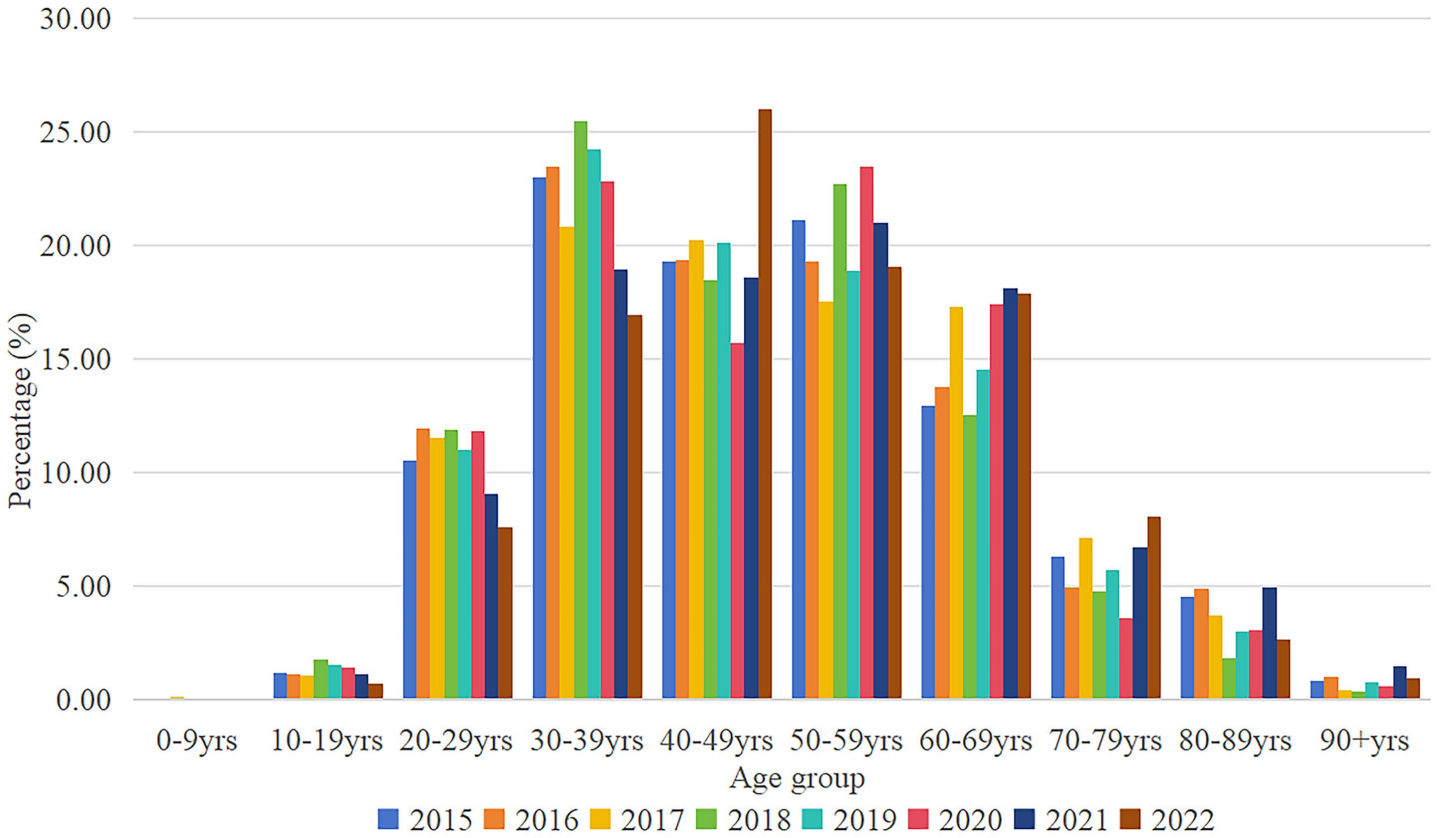
Distribution of CCE_g_ by age groups in Liaoning Province, 2015–2022. CCE_g_, curative care expenditure of gout.

According to Figure 2, it can be known that the age group is divided into 10 age groups according to the stage of 10 years. CCEg share grows rapidly from the age of 20 years, and peaks in 2015–2019 at the age of 30–39 years, the peak occurs in 2020–2021 at the age of 50–59 years, and peaks by the year 2021 at the age of 40–49 years. The structure of the image bar chart shows that gout disease is concentrated among the 30–69 age group, accounting for 70–80% of the total. The ratio of CCEg share from under 50 years old to over 50 years old is in the range of 0.91–1.37 times, with a larger ratio in 2018 and 2019, 1.37 times and 1.32 times, respectively.

**Figure 3:**
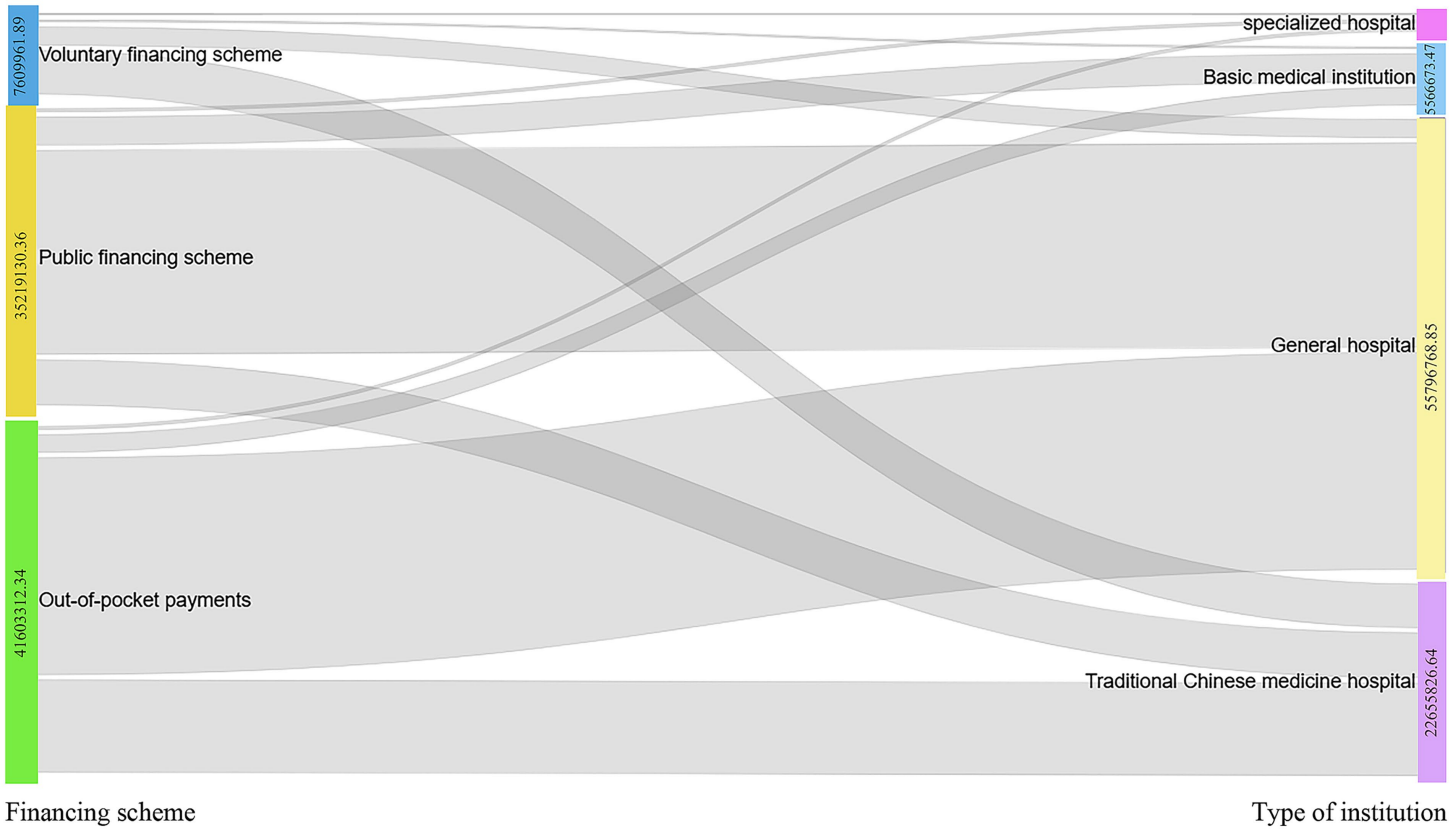
Distribution of average CCEg flows from the three financing modalities to different health care organizations, 2015–2022.

The sankey diagram of this study is classified into general hospitals, traditional Chinese hospitals, specialized hospitals, and primary health care institutions (communal medical service centers, village health centers) (Figure 3). The sankey diagram shows the three financing schemes for average CCEg of Liaoning province during 2015–2022, including funding for public health (CNY 35219130.36), volunteer financing methods (CNY 7609961.89), and out-of-pocket payments (CNY 41603312.34) flowed to general hospitals (CNY 55796768.85), traditional Chinese hospitals (CNY 22655826.64), specialized hospitals (CNY 413135.62), and primary health care institutions (CNY 5566673.47) for specific values. From the composition of the flow chart, the voluntary financing method mainly flows to Chinese hospitals, and both public health financing and self-financing mainly flow towards general hospitals, followed by the flow towards hospitals of Chinese medicine. The main financing of primary care organizations is dominated by out-of-pocket payments (OOP).

The CCE for gout disease in Liaoning province from 2015 to 2022 were CNY 25.32 millions for 2015, CNY 52.08 millions for 2016, CNY 89.42 millions for 2017, CNY 98.27 millions for 2018, CNY 107.46 millions for 2019, CNY 95.96 millions for 2020, CNY 2021 106.77 million, and CNY 116.01 million in 2022. Financing is divided into three types of financing: public financing, voluntary financing, and self-funding, and the share of government financing programs in public financing grows from 48.36% in 2015 to a peak of 50.38% in 2018, and then decreases and then rises again to 42.43% in 2019, and decreases to 21.67% in 2022. Social health insurance in public financing grows from 40.13% in 2015 to a peak of 44.98% in 2017 in the last 8 years, decreases in 2018 to 35.57% in 2019, and gradually decreases to 29.75% in 2020–2022. Out-of-pocket payments grow from 46.45% in 2015 to 2018 58.20%, after decreasing and then growing at 58.17% in 2020 and finally decreasing to 46.17% in 2022. Voluntary financing scheme is the lowest percentage of the three financing methods, with a minimum of 1.39% in 2018 and a maximum value of 11.80% in 2022(Table 3).

**Table 3:** Gout financing cost distribution in Liaoning Province, 2015–2022 [millions (%)].

Year	Public financing scheme			Voluntary financing scheme				Out-of-pocket payments	Total
	Total	Government financing scheme	Social health insurance	Total	commercial insurance	Social donation	Enterprise financing plan		
2015	11.85 (48.36)	2.01 (8.22)	9.83 (40.13)	1.24 (5.20)	0.79 (3.30)	0.04 (0.16)	0.42 (1.74)	12.23 (46.45)	25.32 (100.00)
2016	26.55 (47.14)	2.12 (3.76)	24.43 (43.37)	2.86 (4.92)	1.44 (2.48)	0.08 (0.13)	1.34 (2.31)	22.67 (47.94)	52.08 (100.00)
2017	48.56 (50.38)	5.20 (5.40)	43.35 (44.98)	6.36 (7.78)	4.33 (5.30)	0.03 (0.04)	1.99 (2.44)	34.51 (41.84)	89.42 (100.00)
2018	42.01 (40.42)	10.28 (9.89)	31.73 (30.53)	1.49 (1.39)	0.61 (0.56)	0.17 (0.16)	0.72 (0.67)	54.77 (58.20)	98.27 (100.00)
2019	45.61 (42.43)	7.37 (6.86)	38.24 (35.57)	4.97 (4.58)	4.27 (3.93)	0.05 (0.05)	0.64 (0.59)	56.88 (53.00)	107.46 (100.00)
2020	41.93 (40.36)	7.67 (7.39)	34.25 (32.97)	1.15 (1.47)	0.00 (0.00)	0.33 (0.42)	0.82 (1.05)	52.88 (58.17)	95.96 (100.00)
2021	43.80 (37.66)	9.05 (7.78)	34.75 (29.88)	9.11 (4.83)	7.28 (3.86)	0.16 (0.09)	1.67 (0.88)	53.86 (57.51)	106.77 (100.00)
2022	42.03 (36.23)	7.52 (6.48)	34.51 (29.75)	13.69 (11.80)	12.89 (11.11)	0.12 (0.10)	0.68 (0.59)	60.29 (51.97)	116.01 (100.00)

Liaoning Province 2015–2022 gout disease cost composition in the choice of purchase drug in 2015–2019, 2021 are greater than 96% or so, 2022 accounted for the lowest, only about 80%. By gender, gout disease occurs mainly in males, with the least share being above 88%. Gout mainly occurs in the age of 30–69 years, with a share of more than 76%. The type of medical insurance is concentrated in urban workers’ medical insurance 60–70%, urban and rural residents’ medical insurance between 12 and 32%. The tiers of medical institutions consulted were mainly at the provincial level (65–89%) and 20% and below at the district level, and the types of medical institutions were mainly in general hospitals (70–80%), followed by traditional Chinese medicine hospitals (10–20%) (Table 4).

**Table 4:** Distribution of expenses for gout by different groups, 2015–2022 (million(%)).

	2015	2016	2017	2018	2019	2020	2021	2022
Outpatient/inpatient								
Outpatient	13.37(52.80)	16.74(32.14)	39.99(44.72)	41.00(41.72)	48.89(45.50)	34.63(36.09)	29.19(27.34)	24.87(21.44)
Inpatient	11.95(47.20)	35.34(67.86)	49.43(55.28)	57.27(58.28)	58.57(54.50)	61.33(63.92)	77.58(72.66)	91.14(78.56)
Whether to purchase drug								
Yes	24.40(96.36)	49.37(94.81)	86.23(96.43)	94.80(96.47)	103.82(96.61)	85.64(89.26)	96.14(90.04)	92.87(80.05)
No	0.92(3.64)	2.71(5.19)	3.19(3.57)	3.47(3.53)	3.64(3.39)	10.31(10.74)	10.63(9.96)	23.15(19.95)
Whether to select treatment								
Yes	23.12(91.33)	42.29(81.20)	81.24(90.85)	68.62(69.83)	83.96(78.13)	68.20(71.08)	86.03(80.57)	82.40(71.03)
No	2.20(8.67)	9.79(18.80)	8.18(9.15)	29.65(30.17)	23.50(21.87)	27.75(28.92)	20.74(19.43)	33.62(28.97)
Sex								
Female	2.56(10.12)	3.77(7.23)	6.10(6.82)	4.85(4.94)	5.67(5.27)	6.70(6.98)	7.30(6.83)	13.64(11.76)
Male	22.76(89.88)	48.31(92.77)	83.32(93.18)	93.42(95.06)	101.79(94.73)	89.25(93.02)	99.47(93.17)	102.38(88.24)
Age								
0–29	2.45(9.68)	5.74(11.03)	8.20(9.17)	10.64(10.83)	10.94(10.18)	13.55(14.12)	11.73(10.98)	10.74(9.26)
30–69	19.37(76.49)	40.30(77.37)	70.37(78.69)	80.26(81.67)	85.08(79.18)	75.54(78.73)	81.52(76.35)	91.08(78.51)
≥70	3.50(13.83)	6.04(11.60)	10.85(12.14)	7.37(7.50)	11.44(10.64)	6.86(7.15)	13.52(12.66)	14.20(12.24)
Insurance status								
Urban employees’ basic medical insurance	16.68(65.86)	35.60(68.36)	53.45(59.77)	67.60(68.79)	68.00(63.28)	58.21(60.66)	69.66(65.24)	71.49(61.62)
Basic medical insurance for urban and rural residents	4.63(18.30)	6.40(12.29)	28.69(32.08)	19.24(19.57)	23.53(21.90)	20.90(21.78)	22.77(21.32)	21.35(18.40)
Self-funded	4.01(15.84)	10.08(19.35)	7.29(8.15)	11.44(11.64)	15.93(14.82)	16.85(17.56)	14.34(13.43)	23.18(19.98)
Institution level								
Provincial level	22.29(88.04)	46.67(89.61)	62.71(70.14)	82.73(84.18)	70.88(65.96)	70.07(73.02)	57.72(54.06)	55.83(48.12)
Municipal level	2.36(9.34)	3.88(7.45)	21.85(24.44)	8.96(9.12)	22.31(20.76)	18.62(19.41)	16.38(15.35)	24.00(20.69)
District level	0.08(0.33)	0.14(0.27)	0.59(0.66)	0.22(0.22)	5.63(5.24)	1.00(1.04)	23.11(21.64)	14.75(12.71)
Country level	0.58(2.29)	1.39(2.67)	4.27(4.77)	6.36(6.47)	8.63(8.03)	6.27(6.53)	9.56(8.95)	21.44(18.48)
Institution type								
General hospital	19.99(78.96)	43.15(82.86)	64.22(71.83)	74.62(75.93)	76.83(71.49)	73.09(76.18)	76.35(71.51)	83.32(71.82)
Traditional Chinese medicine hospital	5.28(20.84)	8.74(16.78)	24.78(27.72)	23.40(23.81)	29.94(27.86)	22.10(23.03)	28.89(27.05)	29.81(25.69)
Specialized hospital	0.01(0.01)	0.15(0.29)	0.06(0.07)	0.00(0.00)	0.01(0.01)	0.02(0.02)	0.05(0.05)	0.16(0.14)
Primary medical institutions	0.03(0.12)	0.01(0.01)	0.28(0.31)	0.14(0.14)	0.65(0.61)	0.54(0.56)	1.27(1.19)	2.64(2.27)
Outpatient service organizations	0.02(0.07)	0.03(0.05)	0.04(0.04)	0.04(0.04)	0.03(0.03)	0.20(0.21)	0.21(0.19)	0.08(0.07)
Total	25.32(100.00)	52.08(100.00)	89.40(100.00)	98.20(100.00)	107.46(100.00)	95.95(100.00)	106.76(100.00)	116.01(100.00)

The sample of gout disease in Liaoning province from 2015 to 2022 contains 97,907 cases (Table 5), the sample is based on outpatient (92.14%), purchase drug (56.68%), no treatment (59.37%), male (61.56%), 30–69 years (72.97%), self-funded (57.34%), provincial level (45.03%), general hospital (71.74%), and 2021 (28.78%) played a major role in gout disease. According to the univariate analysis of 2 independent samples and K independent samples test, the outcome of the *p* value is less than 0.05, indicating the existence of remarkable differences in the subgroups of categorical variables in the CCE of gout disease, inpatient, purchase drug, select treatment, male, 30–69, urban employees ‘basic medical insurance, provincial level, traditional Chinese medicine hospital, and 2018 had larger median in various subgroups and the variables with larger median in K independent samples with further pairwise comparisons with *p* value lower than equal to 0.05, which is significantly different, indicating that these variables are associated with having higher CCE for gouty diseases.

**Table 5:** Differences in gout expenses according to the subgroups (*n* = 97,907).

Variables	n (%)	Expenditure median (IQR)	Z/H	*P* value	A two-by-two comparison	*P* value
Outpatient/Inpatient			−144.987^a^	<0.001	None	None
Outpatient	90,213(92.14)	116.60(30.11–329.12)				
Inpatient	7,694(7.86)	7113.03(4956.19–9223.37)				
Whether to purchase drug			−144.715^a^	<0.001		
Yes	55,489(56.68)	281.00(86.90–777.91)				
No	42,418(43.32)	46.00(11.70–173.40)				
Whether to select treatment			−119.913^a^	<0.001		
Yes	39,778(40.63)	334.69(98.64–966.98)				
No	58,129(59.37)	82.20(19.20–238.66)				
Sex			−9.885^a^	<0.001		
Female	37,639(38.44)	130.97(32.96–409.92)				
Male	60,268(61.56)	152.00(36.00–455.84)				
Age			62.272^b^	<0.001		
0–29	19,219(19.63)	138.00(32.96–384.68)			(0–29)-(30–69)	<0.001
30–69	71,445(72.97)	139.84(32.96–436.31)			(0–29)-(≥70)	<0.001
≥70	7,243(7.40)	138.10(33.96–447.05)			(30–69)-(≥70)	<0.001
Insurance status			3608.618^b^	<0.001		
Urban employees’ basic medical insurance	30,893(31.55)	219.00(62.87–677.80)			employees-residents	<0.001
Basic medical insurance for urban and rural residents	10,877(11.11)	156.63(40.36–628.00)			employees-self	<0.001
Self-funded	56,137(57.34)	106.00(22.30–330.04)			residents-self	<0.001
Institution level			16880.032^b^	<0.001	provincial-municipal	<0.001
Provincial level	44,092(45.03)	324.39(100.91–798.73)			provincial-district	<0.001
Municipal level	23,582(24.09)	112.00(33.96–267.38)			provincial-country	<0.001
District level	9,982(10.20)	40.00(9.60–167.88)			municipal-district	<0.001
Country level	20,251(20.68)	45.34(10.99–142.00)			municipal-country	<0.001
Institution type			9157.601^b^	<0.001	district-country	<0.001
General hospital	70,238(71.74)	117.53(28.22–334.77)			general-traditional	<0.001
Traditional Chinese medicine hospital	21,106(21.56)	403.68(119.22–952.71)			general-primary; general-outpatient	<0.001
Specialized hospital	62(0.06)	136.15(44.29–401.25)			traditional-specialize; traditional-primary	<0.001
Primary medical institutions	4,980(5.09)	51.08(19.26–143.98)			traditional-outpatient; specialize-primary	<0.001
Outpatient service organizations	1,521(1.55)	32.00(25.00–60.00)			specialize-outpatient; primary-outpatient	<0.001
Year			2324.703^b^	<0.001	2015–2016;2015–2017;2015–2018	<0.001
2015	3,951(4.04)	160.60(45.34–505.55)			2015–2020;2015–2016;2015–2017	<0.001
2016	5,979(6.11)	158.75(44.60–415.02)			2015–2018;2015–2020;2015–2021	<0.001
2017	6,139(6.27)	209.00(71.00–559.48)			2015–2022;2016–2017;2016–2018	<0.001
2018	6,429(6.57)	247.52(61.67–714.98)			2016–2019;2016–2020;2016–2021	<0.001
2019	9,654(9.86)	180.38(35.30–522.30)			2016–2022;2017–2019;2017–2020	<0.001
2020	13,937(14.23)	226.43(53.70–527.37)			2017–2021;2017–2022;2018–2019	<0.001
2021	28,174(28.78)	95.43(27.54–325.35)			2018–2020;2018–2021;2018–2022	<0.001
2022	23,644(24.15)	98.88(22.03–353.71)			2019–2020;2019–2021;2019–2022;2020–2021	<0.001

a Represents application of Mann–Whitney U test (2 independent samples).

b Denotes non-parametric Kruskal-Wallis H-test (k independent samples). IQR stands for percentile.

Linear regression (Table 6) was used to analyze the factors influencing the cost of gout disease for Liaoning province, and independent parameters included outpatient/inpatient, whether to purchase drug, whether to select treatment, sex, age, insurance status, institution level, institution type, year, without covariates, and high linear correlation among independent variables could explain 60.3% of the cost of gout disease. Based on standardized coefficients (Beta), high gout disease costs were associated with purchase drug, select treatment, basic medical insurance for urban and rural residents, provincial level, traditional Chinese medicine hospital, 2020 were highly associated.

**Table 6:** Multi-linear correlation analysis of cost influencing factors for gouty diseases.

Variable	Unstandardization coefficient		Standardization coefficient	T	Sig
	B(95%CI)	SE	Beta		
Constant	2.501	0.018		135.911	
Outpatient/inpatient					
Outpatient	−1.327	0.007	−0.427	−184.065	<0.001
Inpatient	Reference				
Whether to purchase drug					
Yes	0.567	0.004	0.332	150.138	<0.001
No	Reference				
Whether to select treatment					
Yes	0.369	0.004	0.216	91.078	<0.001
No	Reference				
Sex					
Female	−0.014	0.005	−0.008	−2.663	0.008
Male	Reference				
Age					
0–29	−0.048	0.008	−0.022	−6.121	<0.001
30–69	−0.043	0.007	−0.023	−6.376	<0.001
≥70	Reference				
Insurance status					
Urban employees’ basic medical insurance	0.039	0.004	0.022	9.596	<0.001
Basic medical insurance for urban and rural residents	0.088	0.006	0.033	13.992	<0.001
Self-funded	Reference				
Institution level					
Provincial level	0.424	0.005	0.251	79.708	<0.001
Municipal level	0.213	0.005	0.108	38.928	<0.001
District level	−0.022	0.007	−0.008	−3.135	0.002
Country level	Reference				
Institution type					
General hospital	0.158	0.014	0.085	11.022	<0.001
Traditional Chinese medicine hospital	0.194	0.015	0.095	13.011	<0.001
Specialized hospital	0.242	0.069	0.007	3.504	<0.001
Primary medical institutions	0.098	0.016	0.026	6.228	<0.001
Outpatient service organizations	Reference				
Year					
2015	−0.185	0.011	−0.044	−17.296	<0.001
2016	−0.143	0.009	−0.041	−15.856	<0.001
2017	0.011	0.009	0.003	1.198	0.231
2018	0.081	0.009	0.024	9.408	<0.001
2019	0.037	0.008	0.013	4.833	<0.001
2020	0.152	0.006	0.063	25.822	<0.001
2021	−0.029	0.005	−0.015	−5.958	<0.001
2022	Reference				

R^2^ = 0.603, *p* < 0.001. B is the unstandardized regression coefficient. SE is the standard error. T is the *t*-test value. Sig is the coefficient (P). 95% CI is the 95% confidence interval. Gout expenditures were treated as logarithmic.

In order to check the robustness of the linear regression model, this study replaced whether to select treatment with whether to have a checkup because the variable whether to have a checkup has been previously explored by this research team for its impact on the cost of the illnesses under study (68), and therefore this study also attempted to replace this variable to check the regression model robustness. The results of the multiple linear regression analysis (Table 7) are shown below.

**Table 7:** Multi-linear correlation analysis of cost influencing factors for gouty diseases.

Variable	Unstandardization coefficient		Standardization coefficient	T	Sig
	B(95%CI)	SE	Beta		
Constant	2.362	0.019		123.095	
Outpatient/inpatient					
Outpatient	−1.171	0.008	−0.377	−137.876	<0.001
Inpatient	Reference				
Whether to purchase drug					
Yes	0.526	0.004	0.308	139.17	<0.001
No	Reference				
Whether to have a checkup					
Yes	0.437	0.006	0.189	72.291	<0.001
No	Reference				
Sex					
Female	−0.038	0.005	−0.022	−7.335	<0.001
Male	Reference				
Age					
0–29	−0.007	0.008	−0.003	−0.891	0.373
30–69	−0.026	0.007	−0.014	−3.792	<0.001
≥70	Reference				
Insurance status					
Urban employees’ basic medical insurance	0.028	0.004	0.016	6.868	<0.001
Basic medical insurance for urban and rural residents	0.062	0.006	0.023	9.763	<0.001
Self-funded	Reference				
Institution level					
Provincial level	0.497	0.005	0.294	93.547	<0.001
Municipal level	0.229	0.006	0.116	41.237	<0.001
District level	0.018	0.007	0.006	2.601	0.009
Country level	Reference				
Institution type					
General hospital	0.19	0.015	0.102	13.025	<0.001
Traditional Chinese medicine hospital	0.365	0.015	0.179	24.388	<0.001
Specialized hospital	0.346	0.07	0.01	4.945	<0.001
Primary medical institutions	0.159	0.016	0.041	9.965	<0.001
Outpatient service organizations	Reference				
Year					
2015	−0.181	0.011	−0.043	−16.686	<0.001
2016	−0.135	0.009	−0.039	−14.7	<0.001
2017	0.068	0.009	0.02	7.546	<0.001
2018	0.016	0.009	0.005	1.792	0.073
2019	0.007	0.008	0.002	0.875	0.382
2020	0.125	0.006	0.052	20.882	<0.001
2021	−0.022	0.005	−0.012	−4.553	<0.001
2022	Reference				

R^2^ = 0.591, *P* < 0.001.

The high linear correlation between the independent variables explains 59.1% of the gout costs in Liaoning Province after the robustness check and 60.3% of the gout costs before replacing the variables, which shows that the degree of explanation is still high after replacing the variables, which shows that the model is robust, so the results in Table 6 are credible.

**Figure 4:**
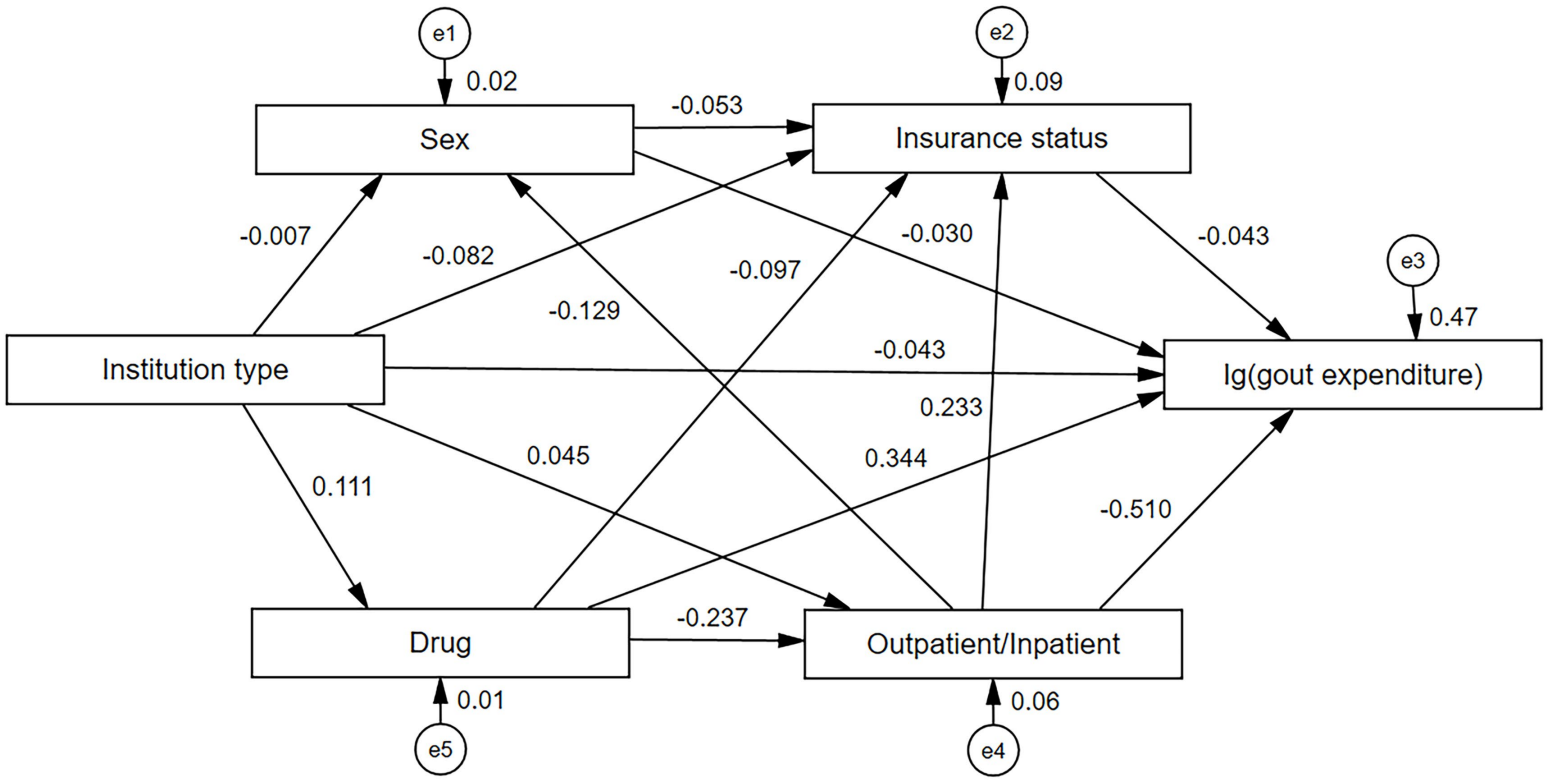
Structural equation modeling and path coefficients of gout disease costs in Liaoning province. Insurance status (in order of actual reimbursement rate): urban workers’ basic medical insurance [actual reimbursement rate = 75.60% (69)] = 1, urban residents’ basic medical insurance (59.70%) (69) = 2, self-funded (0.00%) = 3. Institution type includes general hospitals = 1, traditional Chinese medicine hospitals = 2, specialized hospitals = 3, and primary health care institutions = 4. Drugs include unpurchased drugs = 0, purchased drugs = 1. Gender includes male = 1, female = 2; age: 0–29 = 1, 30–69 = 2, ≥70 = 3. The log of gout outpatient/inpatient expenditure was taken in SEM.

This study explored the direct and indirect influences of variables on gout disease costs (inpatient and outpatient costs) by constructing a SEM (Figure 4). The model in this study had a good fit with c^2^/df = 1.3, RMSEA = 0.002, CFI = 1.000, IFI = 1.000, and TLI = 1.000. Drug had a direct positive effect on gout disease costs (β = 0.344, *P* < 0.001), which was confirmed by insurance status (β = −0.097, *P* < 0.001) and negatively and indirectly through outpatient/inpatient (β = −0.237, *P* < 0.001). Institution type had a direct negative effect on gout disease costs (β = −0.043, *P* < 0.001), a negative indirect effect through sex (β = −0.007, *P* < 0.001), insurance status (β = −0.082, *P* < 0.001), and a positive indirect effect through drug (β = −0.111, *P* < 0.001), outpatient/inpatient (β = 0.045, *P* < 0.001) positive indirect effect.

## 4 Discussion

To the best of our knowledge, this is the first study to analyze the use of and trends in all-age gout funding over an 8-year period from 2015 to 2022 based on the SHA2011 accounting framework. This study provides a comprehensive assessment of the economic burden of gout disease in Liaoning Province and finds that the social and personal economic burden of gout disease is heavy, and the economic burden varies widely across patient populations and healthcare organizations. Outpatient and inpatient costs were imbalanced. The number of gout prevalence in the 8 years studied showed an inverted V-shaped trend, increasing and then decreasing, peaking in 2021. CCE showed an N-shaped change, with 2020 being the low point in the last 5 years. CCE gradually decreased in 2015–2019 at ages 39 years and above, with the greatest value in 2020–2022 at ages 40–59 years. Gout CCE is associated with outpatient/Inpatient, sex, whether to purchase drug, insurance status, institution type, and these correlates contribute to gout cost reduction and policy adjustments.

In our study, we found that the average annual hospitalization burden for gout was CNY 55.33 million, with the share of hospitalized gout increasing from 47.20 to 78.56%, and the average annual outpatient cost was CNY 31.09 million, with a decrease from 52.80 to 21.44%, which is in line with the tendency of the existing studies, where most of the costs were for inpatient hospitalization (71.15%) related to gout (23) and 23.6% was spent on outpatient services (26). We found that descriptive analysis 2015–2021 total cost of hospitalization is the main component of the cost, and with the gradual increase of the year, the share is getting higher and higher. The inpatient expenditure median (CNY 7113.03) was much larger than the outpatient expenditure median (CNY 116.60) in the univariate analysis, and the *p* value < 0.05, this difference was significant, indicating that the mode of visit was inpatient was associated with higher gout costs. The largest value of standardized coefficient (Beta) of multiple linear regression analysis was outpatient (−0.427), indicating that outpatient costs are negatively associated with total costs using inpatient costs as a reference, i.e., the other component of costs, inpatient costs, are positively associated with higher total costs, and the higher the inpatient costs, the higher the total costs. The SEM results showed the largest direct negative effect of outpatient/inpatient log-transformed gout costs, indicating that there is currently a large disparity in outpatient hospitalization costs for gout, and that patients’ choice of hospitalization is associated with high gout costs. There are also studies that agree with our findings that patient hospitalization or outpatient visits are associated with higher gout-related costs (20, 25). Another interesting finding is that the total number of outpatients accounted for 92.14% from 2015 to 2021, while the cost share decreased year by year to 21.44%, and the total number of inpatients accounted for less than 10%, with a cost share as high as 78.56%, which can be speculated that gout patients arriving at hospitalization are very sick, causing a significant disease burden on the patient and the health system to the patient, while outpatient gout patients have symptoms that are milder and the financial burden on the patients is less. This is also consistent with existing research findings that a large percentage of patients with gout need to seek outpatient care (70–72), and only 10–12% of patients are hospitalized for gout-related encounters (73). Therefore there is a need to increase patient adherence to gout primary care guidelines and purinol dosage to reduce the risk of hospitalization from the patient’s point of view, and secondly this study found that a small percentage of patients were hospitalized but the cost was high, therefore it is important to increase the reimbursement rate for hospitalization for gout in order to reduce the burden of care on the patients.

The descriptive statistics of this study analyzed purchase drug expenditure amounted to CNY 24.40million (96.36%) and drug expenditure accounted for more than 80% of the gout cost, which is consistent with the Australian high drug expenditure studies (74, 75). Univariate analysis of purchase drug expenditure median (CNY 281.00) was greater than not purchase drug expenditure median (CNY 46.00) and *p* value <0.05, this difference was significant, indicating that purchase drug was associated with higher gout costs. The multifactor result shows that beta result of purchase drug is 0.332 which is the second highest absolute value which is positive indicating that purchase drug is associated with higher gout cost. SEM result shows positive direct effect coefficient of 0.344 with log gout cost indicating that cost of drug is positively associated with higher gout cost. This study shows that much of the increase in healthcare costs is due to the medication associated with the purchase of medications, this is because strict dietary control can only result in a limited reduction in serum acid levels by 1–2 mg/dL, and the majority of patients need to be dependent on medication (30), and for many patients treatment options may be limited, which may be a reason why medication accounts for a higher cost (76, 77). Gout is a chronic disease that requires long-term medication (78), but some gout medications are not reimbursed by health insurance, which puts tremendous financial pressure on low-income families, the unemployed, and the retired (79). Therefore, the necessary measures to reduce healthcare costs are targeted at reducing drug costs, which can be modeled on the Australian Pharmaceutical Benefits Scheme (PBS) which has subsidized drugs for prescription drugs, effectively reducing costs (80).

In this study general hospital had the highest cost, with the proportion of 70% and above in 2015–2021, followed by Chinese hospitals, with the proportion of 16–27%, and the univariate analysis of Chinese hospitals expenditure median (CNY 403.68) was greater than that of general hospitals expenditure median (CNY 117.53), and *p* value <0.05, *post hoc* comparisons are also significant, indicating that the cost of gout patients in Chinese hospitals is higher than that in general hospitals, and Chinese hospitals are associated with higher gout costs. The multifactorial analysis of Chinese hospitals and general hospitals had a larger beta, with values of 0.095 and 0.085, indicating that Chinese hospitals and general hospitals were associated with higher gout costs with outpatient service organizations as the reference, and the SEM results showed that the institution type had a direct negative effect on the logarithmic transformation of gout costs, with a value of −0.043, indicating that general hospitals, Chinese hospitals, specialized hospitals, and primary health care organizations have lower costs in that order, and general hospitals and Chinese hospitals are associated with higher gout costs. See Table 4 the distribution of the number of patients attending the clinic, the number of patients in general hospitals accounted for 71.74%, hospitals of traditional Chinese medicine accounted for 21.56%, a total of more than 93% of the patients to choose the general hospitals and hospitals of traditional Chinese medicine, large-scale tendency to general hospitals and hospitals of traditional Chinese medicine inevitably result in a higher cost, however, the patient in the primary health care institutions in the routine uric acid-lowering care in order to reduce the financial burden. Therefore, the importance of improving the quality of gout management in primary care is emphasized (81), and it is recommended that, along with other chronic diseases, health-promoting lifestyles be advocated through community public health education (82). Extended consultations by general practitioners when patients first present with gout (83), enhanced monitoring, and instructions to review and follow up patients from time to time (84), education of primary care physicians in the community to optimize the use of uric acid-lowering medications (85), improved knowledge of primary care physicians of the latest treatments and guidelines for gout, and improved skills and treatment regimen patient compliance (19) may prevent gout from progressing to a more severe and more expensive stages, and the burden of disease could be greatly reduced.

The standardized coefficients for urban employees’ basic medical insurance and basic medical insurance for urban and rural residents in the multifactor analysis insurance status were 0.022 and 0.033, and the SEM results showed a direct negative effect on the logarithmic transformation of gout costs, with a value of −0.043, suggesting that medical insurance for urban employees and urban and rural residents has higher gout costs relative to self-funded. The reason for this may be that having health insurance is likely to be a symbol of high socio-economic status (24), and having this economic condition to go to the healthcare institution to cooperate with the treatment and examination is a factor that affects the self-management and control of gout patients, which is consistent with the existing studies (86–88). Conversely, the absence of universal health insurance makes a difference in the treatment of gout, leading to higher complications and mortality (89). In this study, the share of social health insurance expenditure decreased year by year, the share of commercial insurance was seen to increase significantly, the share of OOP increased and then decreased by 46%, and the share of OOP has been around 50%, which shows that the burden of residents’ gout medical care has been greater during these 8 years. In order to reduce the OOP burden for patients, increasing drug co-payments (90), using non-pharmacological approaches (dietary control and lifestyle improvement), increasing subsidies for gout specialty services, popularizing the benefits and disadvantages of over-the-counter medications and benefits effective care modalities (91). In addition to the corresponding recommendations for the macro dimension, corresponding recommendations are also made for the micro level such as family/individual, based on the China Household Finance Survey (CHFS), it is recommended that individuals paying commercial health insurance should choose a flexible contribution period according to their income, which can ensure that more people can make long-term contributions. At the individual and family levels, gout patients are advised to configure health insurance from a scientific and objective perspective, optimize family asset allocation, clearly perceive the importance of insurance, and promote family health insurance participation to maximize family financial benefits and minimize family financial risks (92), in order to cope with the risk of poverty that may be more likely to result from gout. Based on the China Health and Aged Care Longitudinal Study (CHARLS), it is recommended to closely integrate family gout management services with insurance coverage on a family basis, introduce additional gout management services for different gout families, including regular medical checkups, gout counseling, etc., and provide premium discounts or incentives for families with significant improvement in their gout status to motivate them to sustain their gout management, forming a gout management and insurance participation A virtuous cycle of gout management and insurance participation is formed (93). According to the data of the China Family Tracking Survey (CFPS), at the individual level, the out-of-pocket costs of gout patients can be reduced by improving the health of individuals, which can reduce out-of-pocket medical expenses by exercising 1–5 times per week for 31–60 min each time, and the cost reduction is more obvious especially in the middle-aged group (45–59 years old) and the female group (94).

Sex descriptive analysis of male cost share was the largest at 95.06% in 2018, male share was the smallest both at 88.24% from 2015 to 2021, and female maximum cost share was 11.76%. The univariate results were higher and significant (*p* < 0.001) for the median cost for males (CNY 152.00) than the median cost for females (CNY 130.97). Multifactorial analysis with reference to males and standardized coefficient of −0.008 for females, females were associated with lower gout costs and conversely, males were positively associated with higher gout costs. SEM results sex a direct negative effect on log gout costs (−0.030), indicating that males were more costly. Gender is an important influencing factor for gout, which is consistent with existing studies (82, 95–99). There were 60,268 male and 37,639 female patients in this study and the risk of disease in males was 1.6 times that of females, which is lower than the existing studies where the risk of disease in males was 3–4 times that of females (58, 100). The present study showed more significant difference in cost between males and females, with males costing more than 10 times the cost of females, which is higher than existing studies where the burden of gout is 3 times higher in males than in females (101, 102). This is due to hormonal differences and differences due to lifestyle (103), estrogen in women shows slight urinary benefits and adds to uric acid excretion (104, 105), and postmenopausal women have an increased number of patients due to a significant drop on levels of several estrogens which might diminish the renal removal of uric acid leading to an increase in the number of patients (6). Men consume more high-purine meat foods due to social activities or social gatherings (106), have a drinking habit, smoke, or lack of exercise (107, 108), the association of alcohol consumption and meat foods with elevated uric acid levels in serum has been confirmed in relevant studies (109, 110), and the greatest risk for gout is beer, followed by spirits and wine (111), so it is recommended to reduce or abstain from drinking purine-rich beverages.

The research offers further endorsement of public health and primary care initiatives to tackle risk elements and better manage gout. The preventability and treatability of gout suggests that a reduction in the healthcare burden is achievable. New drugs such as febuxostat can be considered for inclusion in local health insurance catalogs in different regions of China to provide more and better choices for the treatment of gout, so that the quality of patient’s survival can be substantially improved, and medical practitioners can be given references for decision-making, so that healthcare resources can be allocated in a more optimal way (112). Strengthening community management and health education for gout patients is conducive to improving life quality of these patients (113). As healthcare professionals, they should give more social support to patients with other forms of health insurance, and also call on the relevant social departments to improve health insurance (88). The whole society should care about the disadvantaged groups of gout patients, and government departments should provide them with more practical help such as improving medical subsidies. Treatment plans with good efficacy but at a cost beyond the patient’s financial ability are difficult for patients to adhere to long-term treatment and are prone to cause disputes, so rheumatologists should fully communicate with rheumatology patients to develop individualized diagnosis and therapy plans based on the patient’s financial situation and acceptance, and maximize the help of patients adhering to standardized treatment (114).

This study has the following limitations, firstly only direct medical costs were calculated in this study, with gout being a disabling primary disease related to loss of employment production (33), other economic impacts including loss of patient productivity, labor shortages, disability costs, transportation costs, as well as absenteeism of family members and companions and transportation costs were not included, therefore the true cost of chronic gout to society as a whole is almost certainly much greater than these estimates. The overall economic impact of recurrent gout attacks from the perspective of employers and society, particularly the indirect costs, requires collection in future studies. Secondly, only patients who were initially diagnosed with gouty illnesses were enrolled, as the reduced renal uric acid excretion and altered purine metabolism in gout can lead to a cumulative burden for many patients, such as hypertension, diabetes, and cardiovascular disease (115), and the present study did not account for the economic burden due to complications and comorbidities resulting from the modified disease. Furthermore, since gout treatment is highly suboptimal, with only one-third to one-half of patients willing to receive uric acid-lowering therapy from healthcare providers, while fewer than one-half of patients adhere to long-term treatment for gout (6, 14), there is a possibility that the direct healthcare costs of gout in this study are underestimated, and that the actual costs will exceed the results of this study.

## 5 Conclusion

Gout disease in Liaoning Province places a heavy financial burden on patients and the health insurance system. Current 2015–2022 gout outpatient and inpatient costs are not reasonably distributed, and the costs are mainly from inpatient costs. Costs are mainly concentrated in the age group of 30–69 years old, financing is mainly concentrated in OOP and public financing programs, and patients’ personal burden is heavy. CCE influencing factors are mainly outpatient/inpatient, institution type, drug, insurance status, sex, and it is recommended to increase the reimbursement rate of gout inpatient cost. The main factors affecting CCE are outpatient/inpatient, institution type, drug, insurance status, sex. It is recommended to increase the reimbursement rate for inpatient gout expenses, to strengthen patients’ adherence to primary care guidelines and purinol dosage, to strengthen the formation of a personalized education and care in the primary care setting led by GPs in collaboration with pharmacists, nurses, community health workers, community advocates to achieve a lowering effect of uric acid, to increase the tilting of the primary care gout policy, to increase the subsidies on gout prescription medicines, to strengthen the addition of gout medicines into the local health insurance directory. Insurance schemes to subsidize most of the costs of general practitioner and specialist consultations and medicines to reduce the financial burden of individual patient visits, and for men to reduce the burden of gout by reducing socialization, consumption of high-purine foods, alcohol consumption, and increased exercise.

## Data Availability

The original contributions presented in the study are included in the article/supplementary material; further inquiries can be directed to the corresponding authors.
